# A Few Words on Bantingism and Obesity

**Published:** 1865-07

**Authors:** Charles Munde

**Affiliations:** Northampton, Mass.


A FEW WORDS ON BANTINGISM AND OBESITY.—*By Charles Munde, M. D., Northampton, Mass.*—Since Mr. Banting published in England his, I might almost say, undeservedly popular letter on the dietetic treatment of obesity, a number of physicians have written on the subject, and attempted to prove to the public that we did not need Mr. Banting to enlighten us on the dietetics, and that we knew everything contained in his letter, long ago, even better than he. Among the numerous, more or less scientific, articles of the kind which I have had occasion to read in different languages, I have, however, found scarcely one which makes the dietetic doctrine more palatable to the non-professional man than does Mr. Banting's letter itself. On the main point they all agree with him, viz., that vegetable diet, containing sugar or starch, produces fat, while animal food does not. From the perusal of all these treatises the public will, therefore, draw no other conclusion than that, in order to become fat, the former, and to become thin, the latter diet is exclusively to be adhered to. To this conclusion I shall now take the liberty of adding a few observations drawn from pratical life.

*First,* In order to remain healthy and vigorous, the system needs for its nutrition a variety of substances, the constituent parts of which resemble, as nearly as possible, those which exist in the body itself. If a person, therefore, use a kind of food, not containing these constituent parts in a correct proportion, of course the nutrition of his body will be performed in an imperfect manner, and he will not only lose flesh, but his health will also suffer, more or less, from such an incomplete regimen.

We therefore not only see those people growing thin who live exclusively on meat, but also those who partake solely of bread or potatoes, notwithstanding the greater proportion of fat-forming ingredients existing in the latter articles. Proofs of this are to be seen in the exclusive vegetarianism of the Hindoos, in the poor German weavers and other inhabitants of mountainous regions, who rarely see a bit of meat, and in prisoners living on bread and water, notwithstanding they lead a sedentary life, which should predispose them to obesity. In thirty years, during which my sojourn in water-cure establishments has given me ample opportunities for making observations on diet, I have found that the majority of corpulent individuals were great consumers of meat; and in most cases, under a suitable course of water treatment and by a simple vegetarian diet, with partial or entire avoidance of animal food, I have succeeded in reducing the weight, and at the same time also in improving the general health of the patient. Thus, but a short time ago, a gentleman from St. Louis, under an exclusive vegetarian diet, was reduced in three months from 295 to 225 pounds, while a fat New York gentleman, under a less strict regimen, lost 20 pounds in a fortnight.

*Secondly.* Nearly all people, or at any rate all who have the means, *eat too much*, and those who live on one kind of food are even obliged to eat too much, in order to draw from their articles of diet, which do not contain the materials composing the body in a correct proportion, a sufficient number of those materials which they would have obtained from a much smaller quantity of mixed food. *He, therefore, who partakes only of one kind of food is compelled to eat more than is healthy for him*, unless he wishes to crave for those substances which his system imperatively demands for the replacement of the daily waste of tissue.

*Thirdly.* It is a well-known fact, that dogs fed exclusively or principally on meat become diseased about the eye, as well as in other parts of the body. This is the case particularly with large dogs, when they have little or no exercise. I myself, within two years have lost two Newfoundland dogs, who had all the physical signs of toxæmia, and who gradually died. In one of them, the first signs of disease appeared in the tail, which lost its hair, became ulcerated and extremely offensive, on which account it was amputated; without relief, however, to the poor fellow, notwithstanding his frequent use of water treatment in the clear mountain stream which flows by my establishment. In the other dog, an abscess formed in the neck, of such size as to make his death unavoidable. No physician will deny that the excessive use of meat will produce also in man a predisposition to inflammatory and putrefactive diseases, inasmuch as he knows that in such disorders the use of animal food is limited or even entirely prohibited. What, then, becomes of the excess of animal food which does not furnish in correct proportion the substances absolutely essential to the nutrition of the body? In my opinion, it produces a diseased condition of the vital fluids, like everything taken in excess, but more so even than a purely vegetable diet. Whether or not this be the case with Mr. Banting, I do not know; I hardly think, however, that he will make an exception to the rule, and that, sooner or later, if he is inclined to be candid, he may be able to write us a less encouraging letter than the one which gave occasion for these remarks.

*Fourthly.* An animal diet does not agree with all persons disposed to corpulency. This especially applies to gouty and plethoric individuals, with whom this kind of food and good living generally must be considered as both the cause and the abettor of their ailments. Recent investigation have shown that the nitrogenous aliments (meat) by reason of their imperfect chemical combination with oxygen, are the chief producers of uric acid, which, accumulating in considerable quantity in the blood, causes the characteristic painful attacks, which do not disappear until by a rigid diet, free perspiration, and other excrementitious processes, the system has become rid of the acid. If this theory (that of Liebig and the Vienna school) be correct (and I have found it confirmed by my own experience), then the advice to corpulent persons disposed to the formation of acids, to eat exclusively or chiefly animal food, is a very poor one; for its adoption would make their principal trouble incurable.

*Fifthly.* If corpulent persons are to be advised conditionally, only, to follow Mr. Banting's example, and if in a country, the inhabitants of which are already inclined to eat much more meat than is good for them, it be a hazardous undertaking to say anything against this particular kind of food, what, then, are those people to do to whom their obesity is not only disagreeable, but also dangerous, especially if there be a predisposition to apoplexy or paralysis? They should eat less and take more exercise, but at the same time adhere to their mixed diet, or, if they be inclined to acidity of the stomach or of the blood, live for a time on a less nutritious, vegetable diet. Such people usually say that they eat extremely little. In most cases this is a delusion, and even those who really eat little, still eat too much—for their particular case. A man can not grow fat on nothing, and, the less a man eats the less adipose tissue will be produced, even if he be unable to take much exercise. This principle, above all, must be observed by every corpulent person. And if he be able, he should take active exercise, such as walking, swimming, gymnastics, fencing, &c., but especially walking. If this be too much for him, he should ride on horseback or in a carriage, practise calisthenics with dumb-bells, clubs, or according to Schreber's method, without any instruments. At the same time, the avoidance of the principal fat-forming aliments, such as sugar, starch, butter, milk, and other highly nutritious articles, or, at least, their very moderate use, and, on the other hand, the adoption of a diet of salads, pickles, oranges, apples, and other fruits, and of less nutritious vegetables, without confining himself too closely to them, are circumstances which will materially aid his purpose.

Cold baths, followed by exercise, also diminish the size of the body, inasmuch as the reaction following them increases the metamorphosis of tissues. If it appear necessary, the temporary administration of some mildly laxative mineral water (Congress water, or a small dose of a solution of sulphate of magnesia, diluted with water) will be of value. However, this should be determined by the scientific and experienced physician, as well as the advisability of a carefully conducted course of water treatment, during which, however, the patient, probably disposed to apoplexy on account of his obesity, should neither be kept wrapped up in a pack for five hours, nor be plunged head over ears into a cold bath. A sojourn of several weeks, not to say months, in a water cure establishment is advisable, if for no other reason than for the diet and the exercise in pure mountain air.

*Sixthly. A steadfast, firm will, and a manly perseverance,* both on the part of the physician, and especially also on that of the patient, are indispensable to an appropriate regulation of the diet and mode of life, generally. "That reminds me of a little story," as our good President Lincoln was wont to say.

A distinguished physician was call into the country to visit a farmer, who, having become wealthy, had, in accordence with his improved worldly condition, gradually accustomed himself to taking more and more of the good things of this life, until, in consequence, he became afflicted with a very inconvenient amount of fat, and with various other ailments, both bodily and mental. After a careful examination, the physician declared that his patient was living too well, and that a gradual return to his former simple and moderate mode of life would remove not only his obesity, but also his corporeal and mental suffering. Half convinced of the truth of the doctor's advice (although he would rather have swallowed a few doses of medicine), he began to express his doubts as to the feasibility of the plan of gradually diminishing the *quantity* of his food, and his ignorance as to the way of doing it. "How many dumplings do you generally eat?" asked the physician (this happened in Saxony, the land of potato dumplings the size of a man's fist). "Twelve," answered the patient. "Well, henceforth," said the doctor, "eat but eleven, and have them made a little larger."

Such is, only too frequently, the advice of the physician on the subject of diet. And, even if he give it in a decided and proper manner, the patient will still find means to interpret his words according to the above example; and thus there is no end to the obesity, the pyrosis, the dyspnœa, the troubled sleep, the irritable temper, the complaints, and—the emetics and laxatives.—*Dental Comoss.*

June 6th, 1865.

